# A Dynamic Mass Redistribution Assay for the Human Sweet Taste Receptor Uncovers G-Protein Dependent Biased Ligands

**DOI:** 10.3389/fphar.2022.832529

**Published:** 2022-02-17

**Authors:** Nicole B. Servant, Mark E. Williams, Paul F. Brust, Huixian Tang, Melissa S. Wong, Qing Chen, Marketa Lebl-Rinnova, Sara L. Adamski-Werner, Catherine Tachdjian, Guy Servant

**Affiliations:** Firmenich, Inc., San Diego, CA, United States

**Keywords:** sweet, taste, receptor, epic, bias ligands, functional selectivity, dynamic mass redistribution, sweetener

## Abstract

The sweet taste receptor is rather unique, recognizing a diverse repertoire of natural or synthetic ligands, with a surprisingly large structural diversity, and with potencies stretching over more than six orders of magnitude. Yet, it is not clear if different cell-based assays can faithfully report the relative potencies and efficacies of these molecules. Indeed, up to now, sweet taste receptor agonists have been almost exclusively characterized using cell-based assays developed with overexpressed and promiscuous G proteins. This non-physiological coupling has allowed the quantification of receptor activity *via* phospholipase C activation and calcium mobilization measurements in heterologous cells on a FLIPR system, for example. Here, we developed a novel assay for the human sweet taste receptor where endogenous G proteins and signaling pathways are recruited by the activated receptor. The effects of several sweet taste receptor agonists and other types of modulators were recorded by measuring changes in dynamic mass redistribution (DMR) using an Epic^®^ reader. Potency and efficacy values obtained in the DMR assay were compared to those results obtained with the classical FLIPR assay. Results demonstrate that for some ligands, the two assay systems provide similar information. However, a clear bias for the FLIPR assay was observed for one third of the agonists evaluated, suggesting that the use of non-physiological coupling may influence the potency and efficacy of sweet taste receptor ligands. Replacing the promiscuous G protein with a chimeric G protein containing the C-terminal tail 25 residues of the physiologically relevant G protein subunit Gα_gustducin_ reduced or abrogated bias.

## Introduction

Two families of G protein-coupled receptors (GPCRs), T1Rs and T2Rs, expressed in specific taste receptor cells on the tongue and palate mediate the taste sensation of sweet-, savory- and bitter-tasting substances. Sweet taste is triggered at the periphery by a pair of GPCRs called T1R2 and T1R3, which function as an obligate heterodimer (Chandrashekar et al., 2006). Agonists such as fructose, sucrose, glucose, aspartame, neotame and saccharin activate the sweet receptor dimer (T1R2/R3) expressed in heterologous cells (Nelson et al., 2001; Li et al., 2002) and deletion of T1R2 or T1R3 in mice eliminates attraction behavior to sweeteners (Zhao et al., 2003; Servant et al., 2020). Umami taste is triggered by a related pair of heterodimeric GPCRs called T1R1 and T1R3 and amino acids such as glutamate (MSG) and aspartate activate the umami receptor dimer (T1R1/R3) expressed in heterologous cells (Li et al., 2002; Nelson et al., 2002). Deletion of T1R1 or T1R3 in mice eliminates attraction behavior to MSG (Zhao et al., 2003; Servant and Frerot, 2021). Bitter-tasting substances are recognized by the T2R family of bitter taste receptors. Human bitter taste receptors for strychnine, salicin, phenylthiocarbamide, saccharin, 6-nitro saccharin, acesulfame K, denatonium and many other bitter substances have been identified (Pronin et al., 2004; Pronin et al., 2007; Meyerhof et al., 2010). T1Rs and T2Rs couple to Gα_gustducin_ in taste receptor cells (Wong et al., 1996). Activation of Gα_gustducin_ in turn activates other effectors such as PLCβ2 and TRPM5 (Zhang et al., 2003) leading to cell depolarization and ultimately taste perception.

**FIGURE 1:**
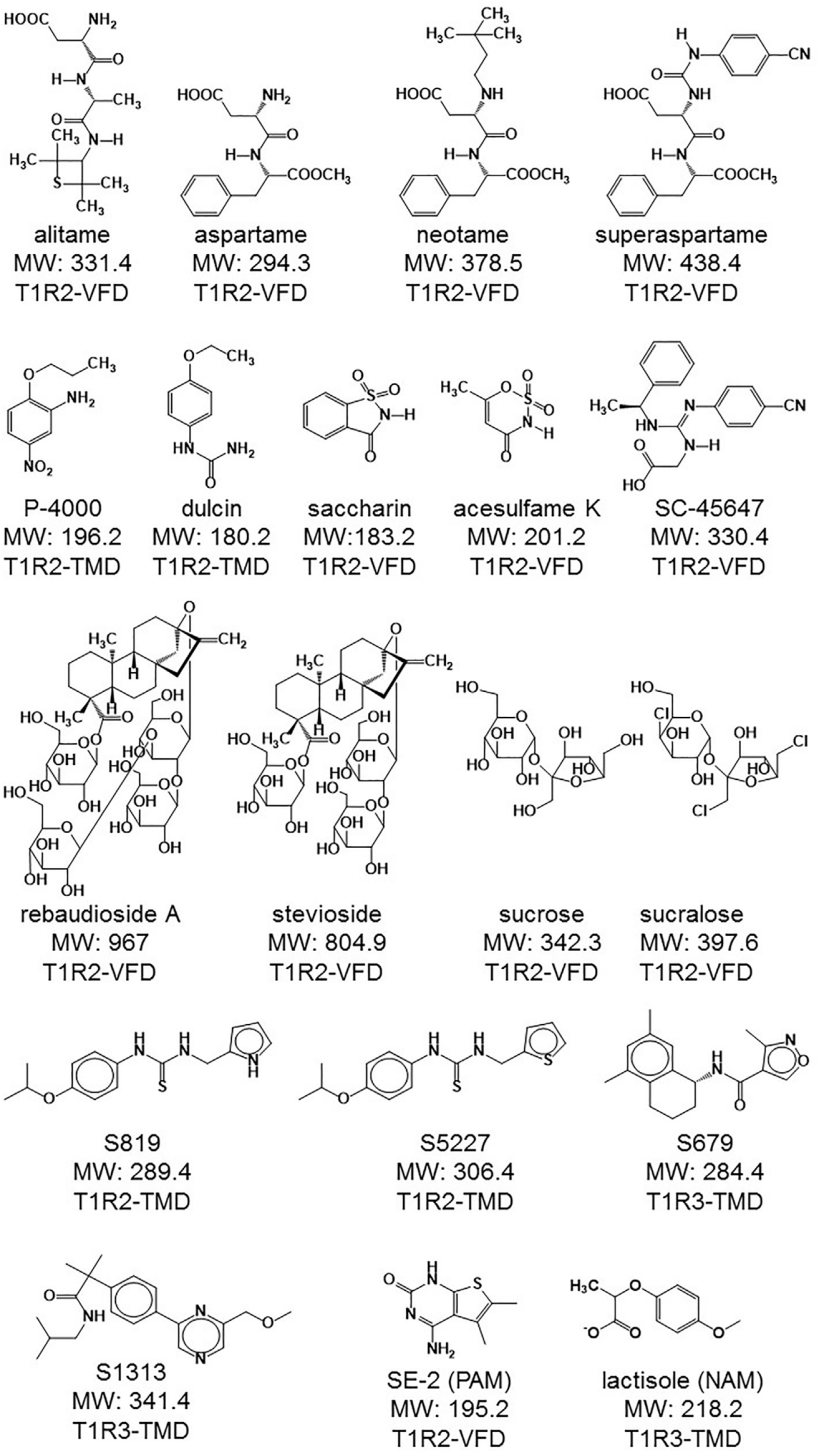
Structures of the different agonists and other modulators used in this study. The respective molecular weight (MW in g/mol) is also indicated as well as the interaction site in the human sweet taste receptor subunits. VFD: Venus flytrap, TMD: transmembrane domain. Interaction site data retrieved from (DuBois, 2016; Servant et al., 2020).

Identification of genes encoding taste receptors has allowed the development of specific cell-based assays that have been used to functionalize the receptors and to characterize the effect of different tastants. Because of the inherent challenges of trying to reproduce the Gα_gustducin_-mediated taste receptor cell signaling pathway in heterologous cells, these assay systems typically use coupling of an overexpressed taste receptor to a non-physiological and promiscuous G proteins, such as Gα_15_, allowing the detection of receptor activation through PLC activation and calcium mobilization (Chandrashekar et al., 2000; Nelson et al., 2001; Bufe et al., 2002; Li et al., 2002; Nelson et al., 2002; Xu et al., 2004). Even if this approach has so far proven successful in identifying new modulators with taste effects (Servant et al., 2010; Zhang et al., 2010; Servant et al., 2011) it is currently not known if various taste receptor ligands, especially those discovered by the screening of compound libraries, can also exhibit functional selectivity, or bias, in the context of coupling to promiscuous and non-physiological G proteins, therefore leading to molecules with an altered pharmacology. The human sweet taste receptor is an ideal model to study the pharmacology and functional selectivity of modulators. This family-C receptor contains multiple binding sites on at least five different domains, allowing for potential allosteric effects between ligands (Jiang et al., 2004; Xu et al., 2004; Jiang et al., 2005a; Jiang et al., 2005b; Winnig et al., 2007; Servant et al., 2020; Behrens, 2021). In addition, more than 100 synthetic and natural molecules have been reported to exhibit a sweet taste (DuBois et al., 2008) (some of them illustrated in Figure 1). In this study we present a descriptive analysis of the effect of 19 different modulators for the human sweet taste receptor, including newly discovered agonists and other modulators, using two different cell-based assays. In one approach, a new assay was developed for the human sweet taste receptor. Ligand-induced dynamic mass redistribution (DMR) measurements were performed on a stable clone exclusively overexpressing the sweet receptor subunits, allowing detection of receptor activation by coupling to endogenous G protein and signaling pathways, and thus, by-passing the need for coupling to overexpressed and promiscuous G proteins. In the other approach, the same cells were transduced with a virus encoding the promiscuous G protein Gα_15_ and calcium mobilization responses to modulators were recorded, as done in other studies (Nelson et al., 2001; Li et al., 2002; Bassoli et al., 2008; Li and Servant, 2008; Servant et al., 2010; Zhang et al., 2010; Bassoli et al., 2014). Relative potencies and efficacies of modulators were ranked and compared. Bias between the two types of assays was also plotted and visualized. A third of the agonists studied exhibited a clear bias towards the calcium mobilization assay (FLIPR assay). This bias was attributed to sweet taste receptor coupling to Gα_15_, as the use of a promiscuous G protein containing the c-terminal tail of Gα_gustducin_ reduced or eliminated the observed bias.

## Materials and Methods

### Materials

The following compounds and toxin were purchased from Sigma-Aldrich (St. Louis, MO, United States): Acesulfame K, aspartame, D-fructose, sucrose, sodium saccharin, monellin, thaumatin, rebaudioside A, lactisole, somatostatin, epidermal growth factor (EGF), SFLLR, Pertussis toxin (PTx), latrunculin A, wortmannin, GW583340, Tyrphostin AG 1478 and GF109203x. Stevioside was from Emperors Herbologists (Jacksonville, FL, United States). P-4000, SC-45647 and superaspartame were gifts from Grant Dubois (the Coca-Cola Company, Atlanta, GA). Sucralose was from Toronto Research Chemicals, Inc. (North York, Ontario, Canada). Alitame (Aclame™) was a generous gift from Danisco (Terre Haute, IN, United States). Neotame was from the NutraSweet Company (Chicago, IL, United States). Dulcin was from Maybridge Chemical Company (Cornwall, United Kingdom). S1P, U1026 and GSK269962 were from Tocris Biosciences (Minneapolis, MN). S819, S5227 (Zhang et al., 2008), S679 (Tachdjian et al., 2009) and S1313 (Tachdjian et al., 2010) were synthesized in house.

### Generation of the R2/R3 U2OS and R2/R3 Gα_16gust25_ U2OS Stable Cell Lines

Untransfected (parental) U2OS cells were cultured at 37°C and 5% CO_2_ in McCoy’s 5A medium (modified, GIBCO#16600) supplemented with 10% fetal bovine serum and penicillin/streptomycin. Plasmids encoding hT1R2 in pEAK10-puromycin and hT1R3 in pcDNA3.1-zeocin were linearized and transfected into U2OS cells followed by dilution and double selection in growth medium plus 0.5 μg/ml puromycin and 100 μg/ml zeocin. Individual colonies were expanded, and half of each colony was transiently transfected with a Gα_16gust25_ expression construct to identify functional clones using sucralose as the agonist in a Ca^2+^ imaging assay. The preserved half (un-transfected with the G protein) of positives clones were further expanded. The clone with the best response and growth characteristics, R2/R3 U2OS, shows functional expression of the sweet taste receptor for greater than 30 passages.

The R2/R3 U2OS clone produced above was subsequently transfected with a linearized plasmid encoding Gα_16gust25_ in pcDNA3-neo. Transfected cells surviving triple selection in growth medium containing 0.5 μg/ml Puromycin (for hT1R2 expression), 100 μg/ml zeocin (for hT1R3 expression) and 500 μg/ml geneticin (Gα_16gust25_ expression) were diluted at various densities to obtain isolated colonies. Individual colonies were expanded, and the presence of functional human sweet receptor and G protein was evaluated in a FLIPR assay using sucralose as the agonist. Selected colonies were further evaluated with a larger panel of agonists. One clone, called L1F2, was used for this study.

### DMR Assays

Parental U2OS cells or cells stably expressing T1R2 and T1R3 (R2/R3 U2OS cells) were seeded on Epic^®^384 microplates (Corning) at a density of 16,000 cells/well in 40 μl of assay media using a multidrop (Thermo Scientific). The plates were incubated in the tissue culture hood for 20 min to allow cells to settle evenly at the bottom of the microplate and further cultured at 37°C in an atmosphere of 5% CO_2_ for 18–24 h. The next day, the cells were subjected to functional analysis using DMR. The lidded Epic^®^ 384 microplate and polypropylene compound plate (Corning) were loaded into the carousel of the Epic^®^ reader, the target plate was washed with DMSO matched D-PBS using the Corning Epic^®^ Liquid Handling Accessory (LHA) and equilibrated in 25 μl of DMSO matched D-PBS for 90 min. After recording the baseline activity for 5–8 min, 25 μl of 2x concentrated solution of agonist or a mixture of agonist and modulator, diluted in D-PBS, was added using the LHA and the DMR responses, which is measured with a shift in light’s resonance wavelength, were monitored for an extra 30 min. When indicated, data for agonist dose-responses were normalized to data obtained with a maximum concentration of sucralose (1 mM) and then fitted and plotted using GraphPad Prism (San Diego, CA) and non-linear regression analysis.

### Evaluation of Pathway Blockers

Blocker stocks (250x) were prepared in DMSO or water and stored as aliquots at −20°C. Just before use, they were diluted to 2X their final concentration in assay buffer (D-PBS) at a final concentration of 0.4% DMSO. Concentrated stock solutions of the agonists somatostatin, carbachol, EGF, and sucralose were made in water or D-PBS while S1P was made up in 50% EtOH. Just before use, the agonists were diluted to 5X their final concentration in D-PBS at a final concentration of 0.4% DMSO. Final agonist concentrations were 10 μM somatostatin, 100 μM carbachol, 100–300 ng/ml EGF, 2 mM sucralose and 1 μM S1P. R2/R3 U2OS cells were plated as described above. The following day cells were washed on the Epic^®^ with D-PBS/0.4% DMSO leaving a residual volume of 10 μl. 10 μl of D-PBS/0.4% DMSO was added to each well by hand followed by 20 μl of 2X pathway blocker. Cell plates were equilibrated on the Epic^®^ for 90 min. After recording the baseline activity for 5 min, 10 μl of 5X agonist was added using the LHA and the DMR responses were monitored for an additional 40 min.

### Generation of Recombinant Baculovirus

A baculovirus shuttle vector derived from pFastBac1 but containing the polylinker and human CMV promoter from pcDNA3.1 was generated as described (Condreay et al., 1999). The Gα_15_ coding sequence was inserted in the HindIII-NotI sites and recombinant virus was generated using the Bac-to-Bac system (Life Technologies). Virus was further amplified by propagation in Sf9 cells grown in suspension using Sf900-II media. Virus titers were determined by plaque assay on Sf9 cells.

### FLIPR Assays

R2/R3 U2OS cells were transduced with recombinant Gα_15_ baculovirus directly in the assay plate as described (Davenport et al., 2009). Briefly, R2/R3 U2OS cells were re-suspended to a final density of 3 × 10^5^ viable cells per mL. Cells were gently mixed with the virus at a multiplicity of infection (MOI) of 5. Using a multidrop, 70 μl per well of the cell and virus mixture was dispensed in a 384-well clear bottom plate (Fisher). After 24 h of incubation in a humidified 37°C tissue culture incubator, trichostatin A was added to a final concentration of 0.3 μM and the cells were incubated for another 18–24 h at 34°C. On the day of the experiment, cells were loaded with 4 μM of the calcium indicator Fluo-4 AM (Invitrogen) in D-PBS for 1 h at room temperature. After washing the cells with D-PBS using a cell washer (BioTek) and a 30-minute rest time, the cell plate and the compound plate were transferred into the FLIPR-Tetra (Molecular Devices). Imaging and data analysis was performed as described (Servant et al., 2010). Alternatively, R2/R3 Gα_16gust25_ U2OS cells were re-suspended at a final density of 2.25 × 10^5^ viable cells per mL and the mixture was dispensed in a 384-well clear bottom plate (Fisher) at 80 μl per well. Loading and the FLIPR assay were run as described above. When indicated, data for agonist dose-responses were normalized to data obtained with a maximum concentration of sucralose (1 mM) and then fitted and plotted using GraphPad Prism (San Diego, CA) and non-linear regression analysis.

### Calculation of Potency and Efficacy Bias and use of an Operational Model

To visualize and assess bias between the two different cell-based assays, we used bias plotting as described (Drake et al., 2008; Peters and Scott, 2009; Gregory et al., 2010; Thomsen et al., 2012; Kenakin, 2015; Montero-Melendez et al., 2015). To calculate relative potency bias between the two assays, we calculated a ratio of the negative logarithm of the EC_50_ (in M) of each agonist to that of sucralose (a sucralose dose-response was performed as a control on the same day) as described: pEC_50_ compound X–pEC_50_ sucralose. This value, that we termed pEC_50_Ratio (sucralose) or pEC_50_R (sucralose), was calculated with EC_50_ values obtained in the two assay systems. This transformation also allowed determination of an average and a standard error for the pEC_50_R (sucralose). The two sets of data (DMR and FLIPR data) were then analyzed with an un-paired *t*-test (α = 0.05). To calculate relative efficacy bias between the two assays, we captured the top asymptote value from each activity-normalized dose-response curve, as provided by the curve fit, calculated an average and a standard deviation (sd) for each agonist and then analyzed the data pairs with an un-paired *t*-test (α = 0.05). Assay bias was also estimated using an operational model (Kenakin et al., 2012; Nagi and Pineyro, 2016). The following steps were followed: 1) Using the PRISM software, we re-analyzed each ligand’s dose-response data obtained in each assay with non-linear regression and the users-defined equation “New Operational Model with TauKa ratios” and retrieved logR values from the fits. 2) Subtracted each ligand’s logR value with that of our standard, the sucralose logR value, obtained from the same data set (each ligand dose-response assay data used in this analysis was accompanied with a sucralose dose-response ran on the same day), producing the ΔlogR value for leach ligand in each assay. 3) Calculated the mean and SEM from ΔlogR replicates (from three to nine independent dose-response analysis, in the two different assays). 4) Calculated ΔΔlogR values by subtracting the averaged ligands’ ΔlogR FLIPR values with the averaged ligands’ ΔlogR DMR values. 5) Determined if the 95% confidence interval of the ΔΔlogR values include 0. Confidence intervals were calculated from ΔΔlogR SEM values adjusted using factors obtained in the t-student distribution table at (t_*n-1*_; _0.975_), with *n* values corresponding to the sum of the number of observations in each group, −1, as described in (Kenakin et al., 2012).

## Results

**FIGURE 2:**
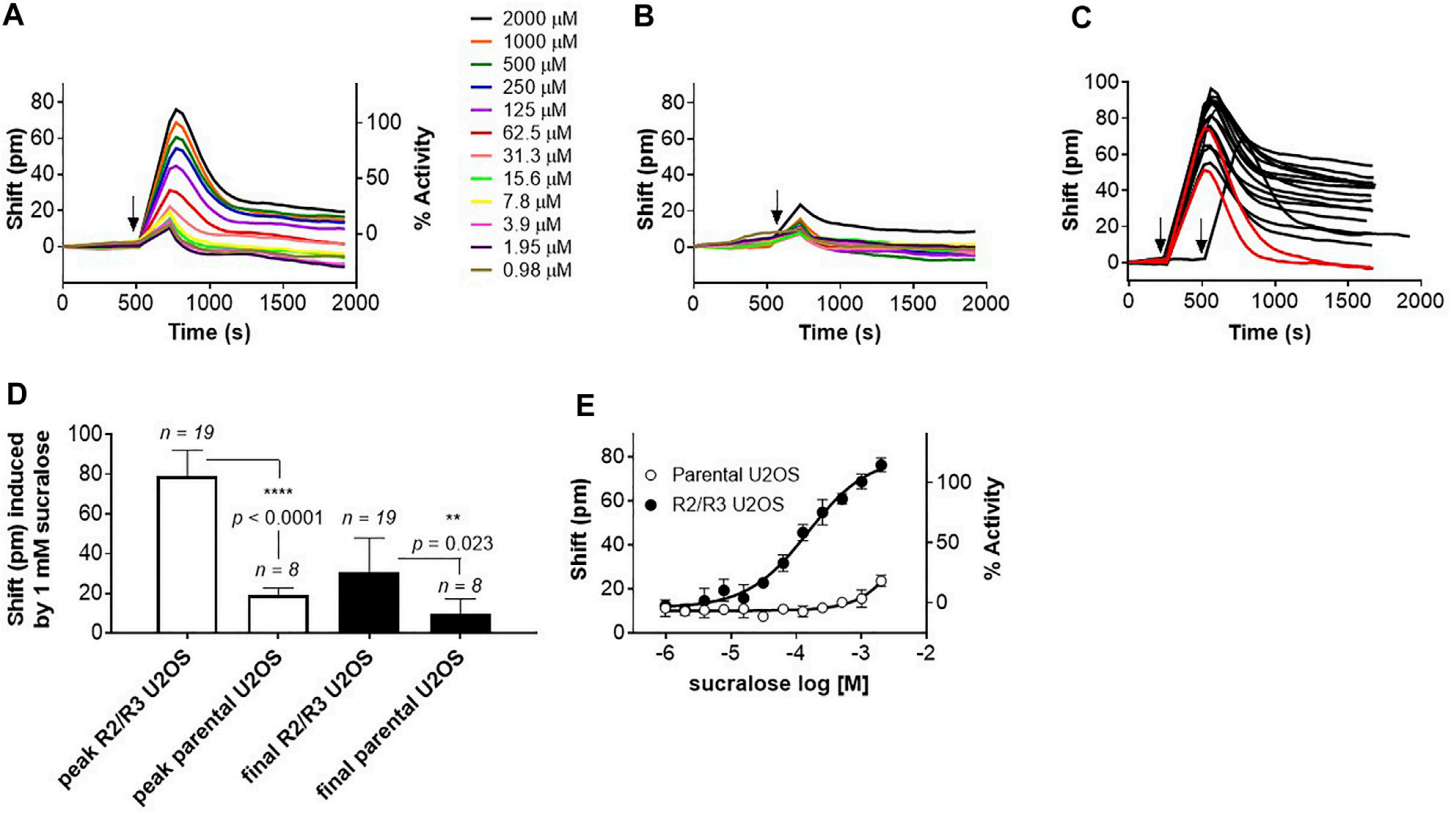
Development of a DMR assay for the human sweet taste receptor. **(A)** Application of increasing concentrations of sucralose onto R2/R3 U2OS cells cause a positive DMR signal. Arrow indicates time of application. **(B)** Application of increasing concentrations of sucralose onto parental U2OS cells does not cause a comparable positive DMR signal. Arrow indicates time of application. **(C)** Kinetics of sucralose (1 mM)-induced DMR responses over a period of 3 months. 19 independent experiments were conducted during that period. In 2 of these experiments (red traces) the DMR responses returned to background levels while in the remaining experiments they stayed above background levels. Arrows indicate time of application. **(D)** Determination of assay window robustness at the peak or final DMR responses. A maximum concentration of sucralose (1 mM) was used to stimulate either R2/R3 U2OS cells or parental U2OS cells (number of independent experiments are indicated on the bar graph). Peak and final DMR responses were recorded and averaged. Data shows that measurement at the peak response provides a better assay window (unpaired, *t*-test, α = .05). **(E)** Representative sucralose dose-response analysis on R2/R3 U2OS cells and parental U2OS cells. Dose-response is representative of several independent experiments and each data point corresponds to an average and standard deviation of a triplicate determination. Averaged potency value for sucralose is summarized in Table 1.

The hT1R2 and hT1R3 sweet receptor subunits were introduced in U2OS cells and a stable clone was used to develop a DMR assay (see *Materials and Methods*). Upon addition of the high potency agonist sucralose onto the R2/R3 U2OS cells, a well-defined positive DMR response was detected (Figure 2A). The sucralose effect was dose-dependent and could not be picked up on the parental U2OS cell line (Figures 2A,B). The shape of the DMR kinetics on R2/R3 U2OS cells was consistent, usually producing a peak response at ∼4 min post-stimulation, and then subsiding and stabilizing in the ensuing 20 min to a sustained response with about one half to one third of the peak response magnitude (Figures 2A,C). In just a few occasions, the positive DMR responses fully returned to background levels relatively rapidly following the peak response (red traces in Figure 2C; in 2 out of 19 experiments performed with sucralose over a period of 3 months). To determine how to precisely quantify the effects of agonists, both the peak and sustained final responses were recorded on the R2/R3 U2OS cells and compared to the receptor-independent responses obtained with the parental U2OS cells. Measuring the peak DMR response turned out to provide a much more robust assay window than measuring the final and sustained DMR response (Figure 2D). For this reason, subsequent data depicted in this report was generated with the peak DMR responses, as reported in other DMR studies with different GPCRs (Schrage et al., 2013; Malfacini et al., 2018; Ruzza et al., 2018). Under these conditions, sucralose generated an EC_50_ of 163 μM in this assay (Figure 2E; Table 1) (pEC_50_ 3.825 ± 0.174) and only the highest concentration of sucralose (≥2 mM) produced a smaller non-specific effect on the parental U2OS cells [typically around 20–40 pm (pm), Figures 2B,E].

**TABLE 1:** Summary of potency and efficacy values for sweet taste receptor agonists used in this study. Agonist Emax values were compared to that of sucralose in the same assay and analyzed by an unpaired, two-tailed *t*-test (α = .05).

Sweetener	Activity in DMR assay							Activity in FLIPR assay						
	EC50 (μM)	pEC50	SD	*N*	Emax	SD	*N*	EC50 (μM)	pEC50	SD	*N*	Emax	SD	*N*
S819	0.7	6.196	0.114	4	143*	9	4	0.07	7.128	0.041	3	181****	6	3
S679	1.3	5.914	0.147	7	133	12	3	0.7	6.145	0.115	4	159****	20	4
S1313	1.5	5.822	0.120	7	130	8	3	0.7	6.177	0.159	4	170****	22	4
S5227	2.2	5.685	0.184	4	128	9	4	0.3	6.475	0.059	3	177****	8	3
P-4000	3.8	5.423	0.097	7	102	7	3	2.2	5.674	0.144	3	164****	20	3
SC-45647	4.3	5.388	0.146	10	97**	18	6	2.3	5.642	0.095	4	98**	4	4
Neotame	7.7	5.137	0.156	4	99*	7	4	1.6	5.815	0.077	4	116****	6	4
Superaspartame	9.8	5.023	0.128	4	109	8	4	4.7	5.335	0.053	4	105	1	4
Dulcin	33	4.513	0.166	9	57****	7	9	23	4.637	0.061	4	87****	5	4
Alitame	37	4.488	0.231	4	106	27	4	20	4.713	0.054	4	100	4	4
rebaudioside A	57	4.293	0.238	7	103	26	3	29	4.538	0.066	4	106	6	4
Stevioside	127	3.943	0.212	7	141	60	3	51	4.300	0.079	4	102	5	4
Sucralose	163	3.825	0.174	22	122	19	22	72	4.159	0.115	22	104	4	22
Saccharin	411	3.482	0.402	6	145	47	3	241	3.743	0.351	4	90****	**5**	4
Aspartame	910	3.113	0.246	6	143	17	3	354	3.453	0.043	4	106	6	4
acesulfame K	1,221	3.043	0.352	6	122	78	3	253	3.601	0.069	4	73****	7	4

****p < .0001, **p < .01, *p < .05.

**FIGURE 3:**
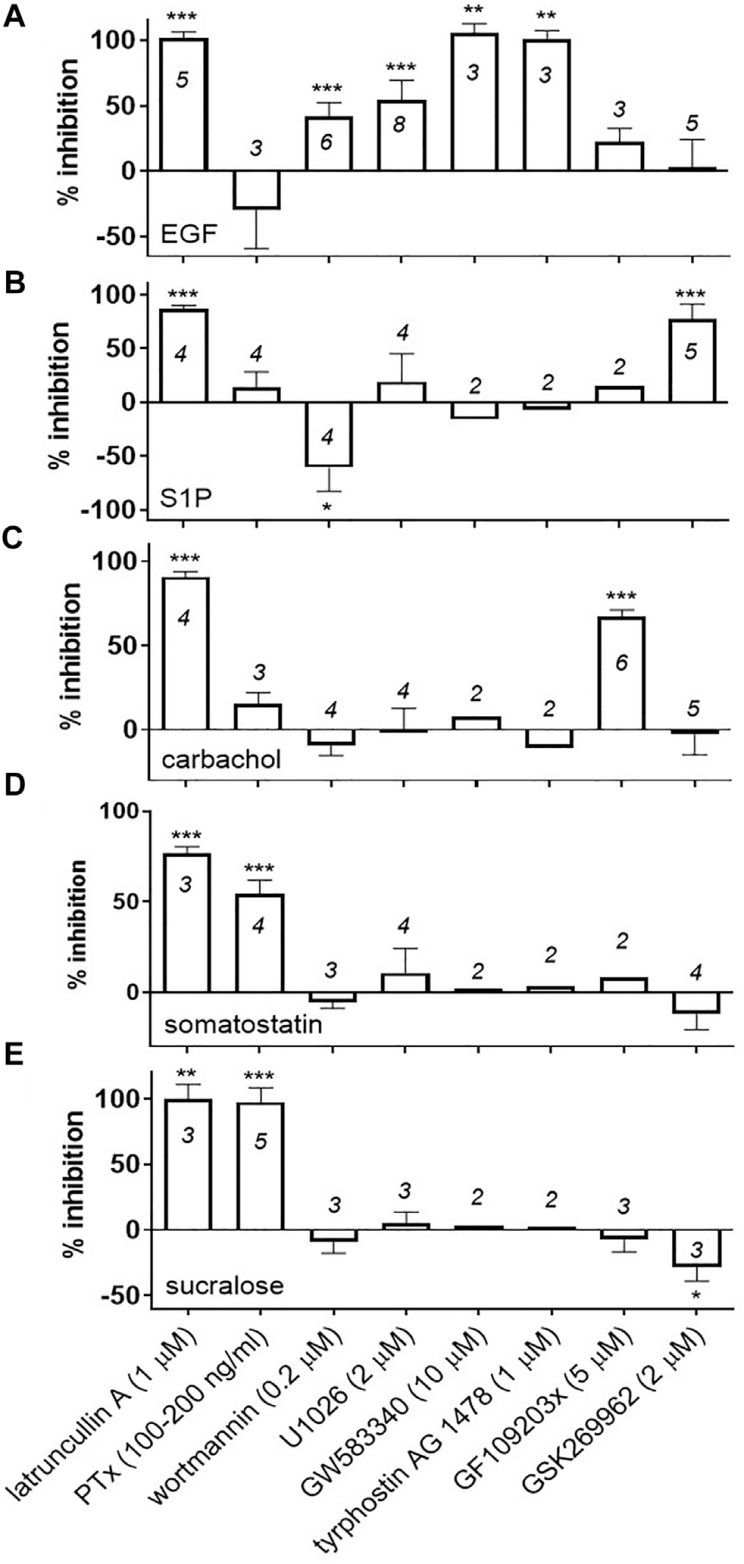
Effect of pathway blockers on agonist-mediated DMR responses in R2/R3 U2OS cells. Cells were equilibrated at room temperature for 90 min in the Epic^®^ reader with the indicated concentrations of pathway blockers or latrunculin A. In the case of PTx, cells were incubated over night at 37°C and on the day of the experiment PTx was replaced with buffer and cells were equilibrated in the Epic^®^ reader as described above. A 5x-concentrated stock of agonist was then added onto the cells and DMR responses were recorded as described in *Materials and Methods*. Final concentrations of agonist were 2 mM sucralose **(E)**, 10 μM somatostatin **(D)**, 100 μM carbachol **(C)**, 1 μM S1P **(B)** and 100–300 ng/ml EGF **(A)**. Two to eight independent experiments were conducted with each blocker (number of independent experiments are indicated on the bar graph). For the experiments conducted with sucralose, a % inhibition was calculated for each pathway blocker using an assay window defined by the peak DMR value of the sucralose response alone measured on R2/R3 U2OS cells (0% inhibition) and the sucralose response measured on parental cells (100% inhibition). Alternatively, for the remaining agonists a % inhibition was calculated for each pathway blocker using an assay window defined by the peak DMR value of the agonist response alone measured on R2/R3 U2OS cells (0% inhibition) and a buffer stimulation on R2/R3 U2OS cells (100% inhibition). A mean and standard deviation was calculated and a one sample *t*-test (α = .05) was conducted for each blocker with N ≥ 3, determining if the mean inhibition was different than 0% (***p < .001, **p < .01, *p < .05). Representative DMR traces are shown in Supplementary Figure S1.

Next, we used pharmacological inhibitors or toxins to find out which signaling pathways contribute to the DMR responses of the sweet taste receptor in U2OS cells and, as control experiments, we also evaluated the effects of the same inhibitors on different endogenous receptor pathways. An intact actin cytoskeleton was absolutely required to generate optimal receptor modulator responses in this assay. Latrunculin A (1 μM), a toxin that sequesters G-actin and prevents it from polymerizing into F-actin (Servant et al., 2000), disrupted the DMR responses to sucralose, somatostatin, the muscarinic receptor agonist carbachol, the bioactive lipid mediator sphingosine-1-phosphate (S1P) and the receptor tyrosine kinase agonist epidermal growth factor (EGF) (Figure 3; representative effects of pathway blockers on DMR responses are depicted in Supplementary Figure S1). PTx, which ADP-ribosylates members of the Gα_i/o_ family and uncouples them from activated receptors (Fields and Casey, 1997), completely inhibited the sucralose response (Figure 3E) and also significantly weakened the somatostatin response (54 ± 8% inhibition; mean ± sd; *n* = 4) (Figure 3D) while having little or no inhibitory effects on the carbachol response (16 ± 7% inhibition; mean ± sd; *n* = 3) (Figure 3C), the S1P response (14 ± 14% inhibition; mean ± sd; *n* = 4) (Figure 3B) or EGF response (−29 ± 29% inhibition; mean ± sd; *n* = 3) (Figure 3A). Gα_i/o_ protein mediated signaling pathways are known to regulate actin cytoskeleton remodeling (Rickert et al., 2000). Therefore, we investigated some of the key players known to feed in this specific pathway such as phosphatidylinositol 3-kinases (PI3K), MAPK kinase, receptor tyrosine kinases, protein kinase C (PKC) and a small GTPase effector, Rho Kinase (ROCK) (Rickert et al., 2000). While maximum concentrations of wortmannin (200 nM) and U0126 (2 μM), inhibitors of PI3K and MEK, respectively, blocked the EGF response by 42 ± 10% (mean ± sd, *n* = 6) and 54 ± 15% (mean ± sd, *n* = 8) (Figure 3A), they had no statistically significant effects on the sucralose response, the somatostatin response and the carbachol response (Figures 3C–E, respectively). Noticeably, the same concentration of wortmannin enhanced the S1P response in R2/R3 U2OS cells (61 ± 22% enhancement; mean ± sd; *n* = 4) (Figure 3B). U0126 did not significantly inhibit the S1P response (19 ± 26% inhibition; mean ± sd; *n* = 4) (Figure 3B). Conversely, the PKC inhibitor GF109203x (5 μM) significantly inhibited the carbachol response by 67 ± 4% (mean ± sd, *n* = 6) (Figure 3C) but had little or no effect on the sucralose response (−7 ± 10 % inhibition; mean ± sd; *n* = 3), the somatostatin response (average of 8% inhibition, two independent experiments, 15 and 1% inhibition), the S1P response (average of 15% inhibition, two independent experiments, 15% inhibition obtained in both experiments), or the EGF response (22 ± 11% inhibition; mean ± sd; *n* = 3) in the same cells (Figures 3A,B,D,E respectively). Transactivation of receptor tyrosine kinases, such as the EGFR, by GPCRs has been proposed as a mechanism leading to actin remodeling (Daub et al., 1996; Schraufstatter et al., 2002; Calandrella et al., 2005). Treatment of R2/R3 U2OS cells with two of the EGFR inhibitors evaluated, GW583340 (10 μM) and tyrphostin (1 μM), abolished the EGF response (Figure 3A) but had no effect on the sucralose response (GW583340, average of 3.1% inhibition, −1 and 7% inhibition in two independent experiments; tyrphostin, average of 2.6% inhibition, 0% and 5% inhibition in two independent experiments), the somatostatin response (GW583340, average of −0.3% inhibition, −12% and 11% inhibition in two independent experiments; tyrphostin, average of 3.1% inhibition, −11% and 7% inhibition in two independent experiments), the carbachol response (GW583340, average of 7.6% inhibition, 10% and 5% inhibition in two independent experiments; tyrphostin, average of −10.8% inhibition, −2% and −20% inhibition in two independent experiments), and the S1P response (GW583340, average of −16% inhibition, −17% and −14% inhibition in two independent experiments; tyrphostin, average of −7% inhibition, −18 and 3% inhibition in two independent experiments) (Figures 3B–E, respectively). Small GTPases of the Rho-subfamily and their effectors likewise regulate ligand-induced F-actin remodeling and are known to act downstream of GPCRs and receptor tyrosine kinases (Hall, 1998). Rho-associated protein kinase (ROCK) is activated by the small GTPase RhoA which usually leads to stress fiber formation (Mackay and Hall, 1998). Its inhibitor, GSK269962 (2 μM), significantly blocked the S1P response (77 ± 14% inhibition; mean ± sd; *n* = 5) (Figure 3B). However, GSK269962 had no inhibitory effect on the sucralose response, but instead, enhanced the response by 28 ± 11% (mean ± sd; *n* = 3) (Figure 3E). The somatostatin, carbachol and EGF responses were not affected by this inhibitor (Figures 3A,C,D respectively). Collectively these results reveal that sweet taste receptor positive DMR responses in U2OS cells are mediated through Gα_i/o_ proteins and require an intact actin cytoskeleton. Gα_i/o_ specific pathways and recruited second messengers leading to the sucralose-induced positive DMR responses still need to be identified.

**FIGURE 4:**
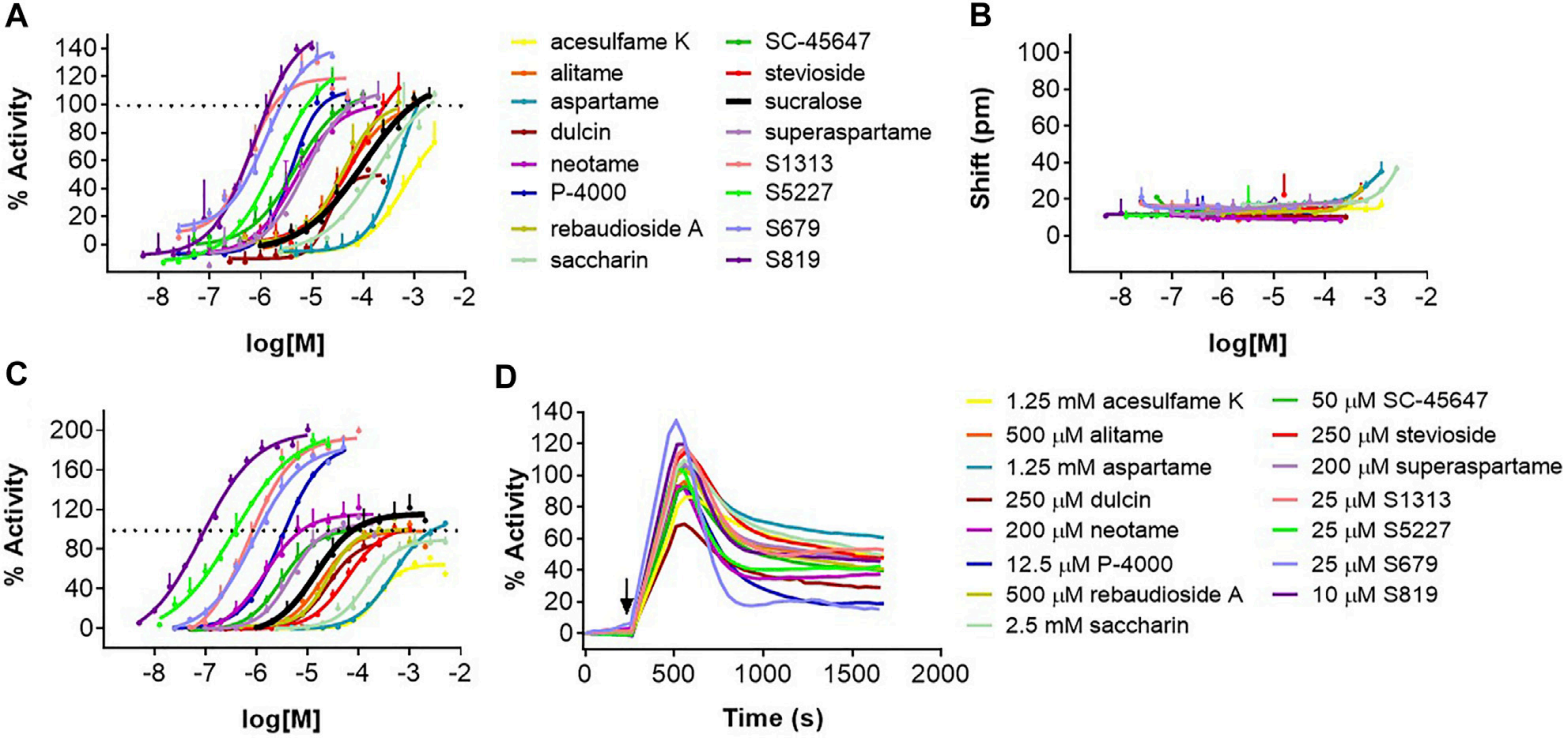
Dose-response analysis of sweet taste receptor agonists in the DMR and FLIPR assays. Agonists were evaluated in the DMR assay with R2/R3 U2OS cells **(A)** and U2OS parental cells **(B)** and in the FLIPR assay with R2/R3 U2OS cells transduced with a Bacmam virus encoding Gα_15_ **(C)** as described in *Materials and Methods*. **(D)** Kinetics of indicated agonist responses in the DMR assay using R2/R3 U2OS cells. Dose-responses are representative of several independent experiments and each data point corresponds to an average and standard deviation of a triplicate determination.

In following experiments, we evaluated a panel of known high potency agonists, carbohydrate sweeteners (Figure 1), and novel sweet taste receptor agonists. We performed dose-response analysis of each individual molecule in the DMR assay and evaluated the same molecules using R2/R3 U2OS cells overexpressing the promiscuous G protein Gα_15_ for their ability to promote calcium mobilization (FLIPR assay; see *Materials and Methods*). The goal was to determine and compare the relative rank order of potency and relative efficacies obtained in the two different assays. About 80% of the agonists evaluated produced specific responses with EC_50_s < 200 μM in the DMR assay (Figures 4A,B; Supplementary Figure S2; Table 1). These included the high potency agonists of the guanidinoacetic acid family such as SC-45647, the aspartame analogues such as alitame, neotame and superaspartame, the nitroaniline P-4000, the phenyl urea dulcin and the terpenoid glycosides including rebaudioside A and stevioside. Overall, these agonists exhibited similar rank order of potency in the FLIPR and DMR assays (Figures 4A,C; Supplementary Figures S3; Table 1). For the majority of the depicted agonists, the DMR EC_50_ values were about 2–3 times higher than those obtained in the FLIPR assay (Table 1). The most potent agonists characterized in this study were the ones identified by high throughput screening in a human sweet taste receptor assay followed by assay-guided chemical optimization. S819 (Zhang et al., 2008) and its analog S5227 correspond to optimized agonists of the thiourea series. S679 corresponds to an optimized agonist of the tetralin amide series (Tachdjian et al., 2009) while S1313 corresponds to an optimized agonist of by bi-aryl series (Tachdjian et al., 2010; Figure 1). These newly discovered agonists activated the sweet receptor with potencies of 0.7–3 μM in the DMR assay using R2/R3 U2OS cells (Figure 4A; Table 1). S819 and S5227 were much more potent in the FLIPR assay with EC_50_s of 0.07 and 0.3 μM, respectively with values about 10 times lower than their EC_50_s in the DMR assay (Figure 4C; Table 1). Moreover, while S5227 was less potent than S679 and S1313 in the DMR assay, the reverse was true in the FLIPR assay. The remaining agonists evaluated in the DMR assay produced EC_50_s > 200 μM. These included saccharin, aspartame and acesulfame K in descending order of potency (Figures 4A,B; Table 1). The same agonists were 2–4 times more potent in the FLIPR assay (Figure 4C; Table 1). Saccharin and acesulfame K are thought to exhibit both agonistic (at lower concentrations, first binding site) and antagonistic properties (at higher concentrations, second binding site) on the sweet taste receptor when using promiscuous G proteins (Galindo-Cuspinera et al., 2006). This is probably why they failed to fully activate the sweet receptor in the FLIPR assay (Figure 4C and Emax values in Table 1). We could not detect this effect in the DMR assay (Table 1). Of note, however, is that these agonists, also including aspartame and stevioside, did not always produce dose-response curves with a well-defined top asymptote in the DMR assay (see Supplementary Figure S2), resulting in different efficacy values (Emax) from experiment to experiment as reflected by the relatively higher Emax standard deviation values (Table 1). S819 behaved as a superagonist in the DMR and FLIPR assays, producing responses significantly greater than that obtained with a 1 mM sucralose concentration (where the sucralose dose-response curve’s top asymptote plateaued at around 122% in the DMR assay and at 104% in the FLIPR assay) (Figures 4A,C; Table 1). The other potent agonists S5227, S679, and S1313 also behaved as superagonists relative to sucralose in the FLIPR assay (Table 1). While there was a trend indicating a higher level of activity for these agonists relative to sucralose in the DMR assay, the differences were not statistically significant (Table 1). Qualitatively, the agonists evaluated in this study exhibited similar DMR kinetics at maximal concentration (Figure 4D).

Even though we could reliably detect the activity of every high potency agonist studied (as shown above), we could not detect any specific DMR responses for carbohydrate sweeteners in R2/R3 U2OS cells. As shown in Supplementary Figure S4, sucrose (table sugar) interfered directly with the biosensor, producing high signals even in the absence of cells. Likewise, sucrose produced significant DMR responses on the parental U2OS cells at all concentrations evaluated. No significant sucrose-induced change in DMR in R2/R3 U2OS cells could be detected when normalizing the responses for the background detected in U2OS parental cells (Supplementary Figures S4H,I). Similar results were obtained with D-fructose (results not shown). On the other hand, sucrose and D-fructose responses could be readily detected in the FLIPR assay performed on R2/R3 U2OS cells transduced with Gα_15_ (Supplementary Figure S5; Servant et al., 2020).

**FIGURE 5:**
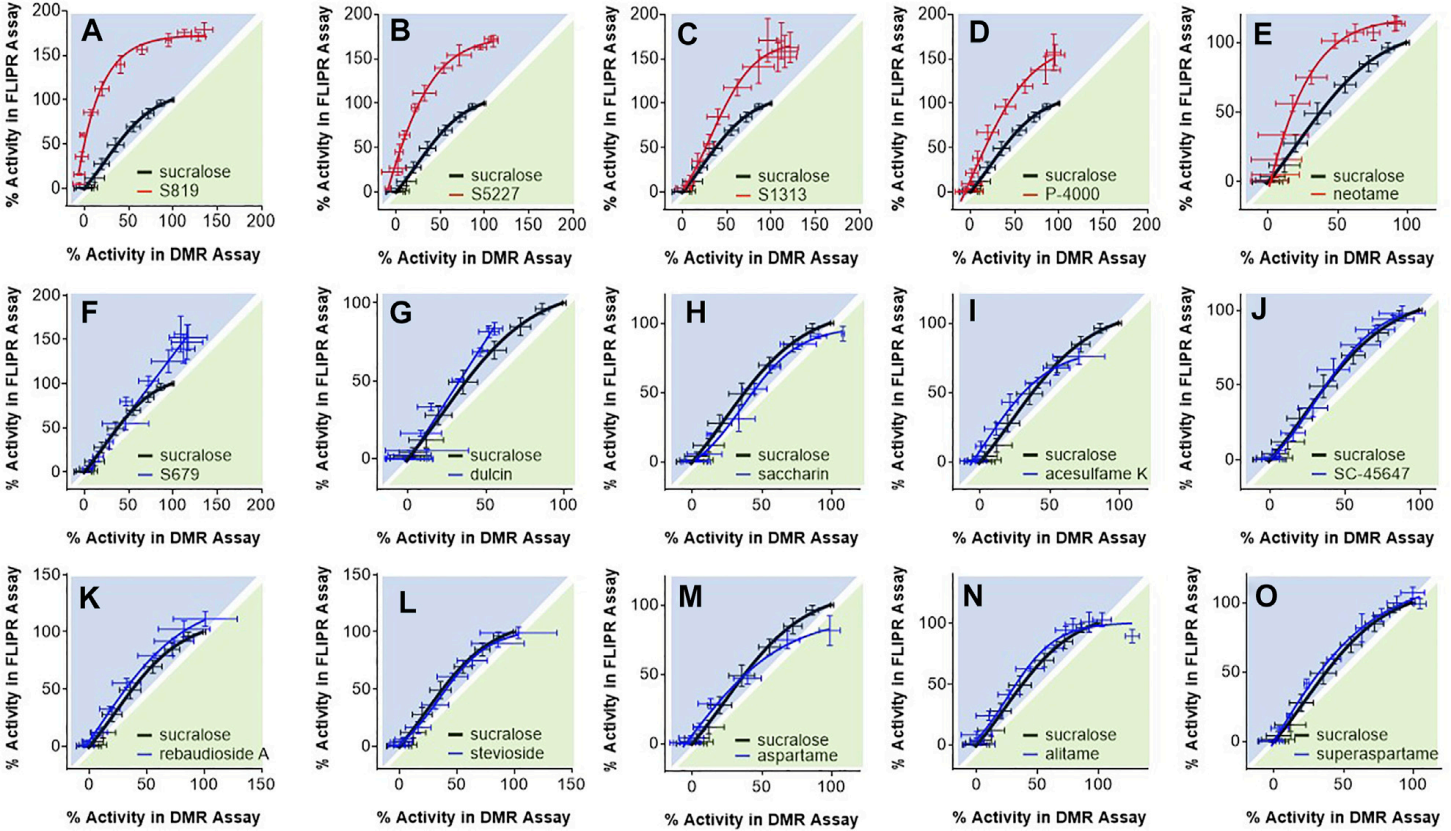
Bias plotting analysis. Depicted agonists [panels **(A–O)**] were evaluated at equimolar concentrations in the DMR assay with R2/R3 U2OS cells and in the FLIPR assay with R2/R3 U2OS cells expressing Gα_15_. Assay data was normalized to responses obtained with 1 mM sucralose and resulting activity values were plotted on the same graph. Each data point corresponds to an average and standard deviation from values generated in 3 to 21 independent experiments performed in triplicate.

**FIGURE 6:**
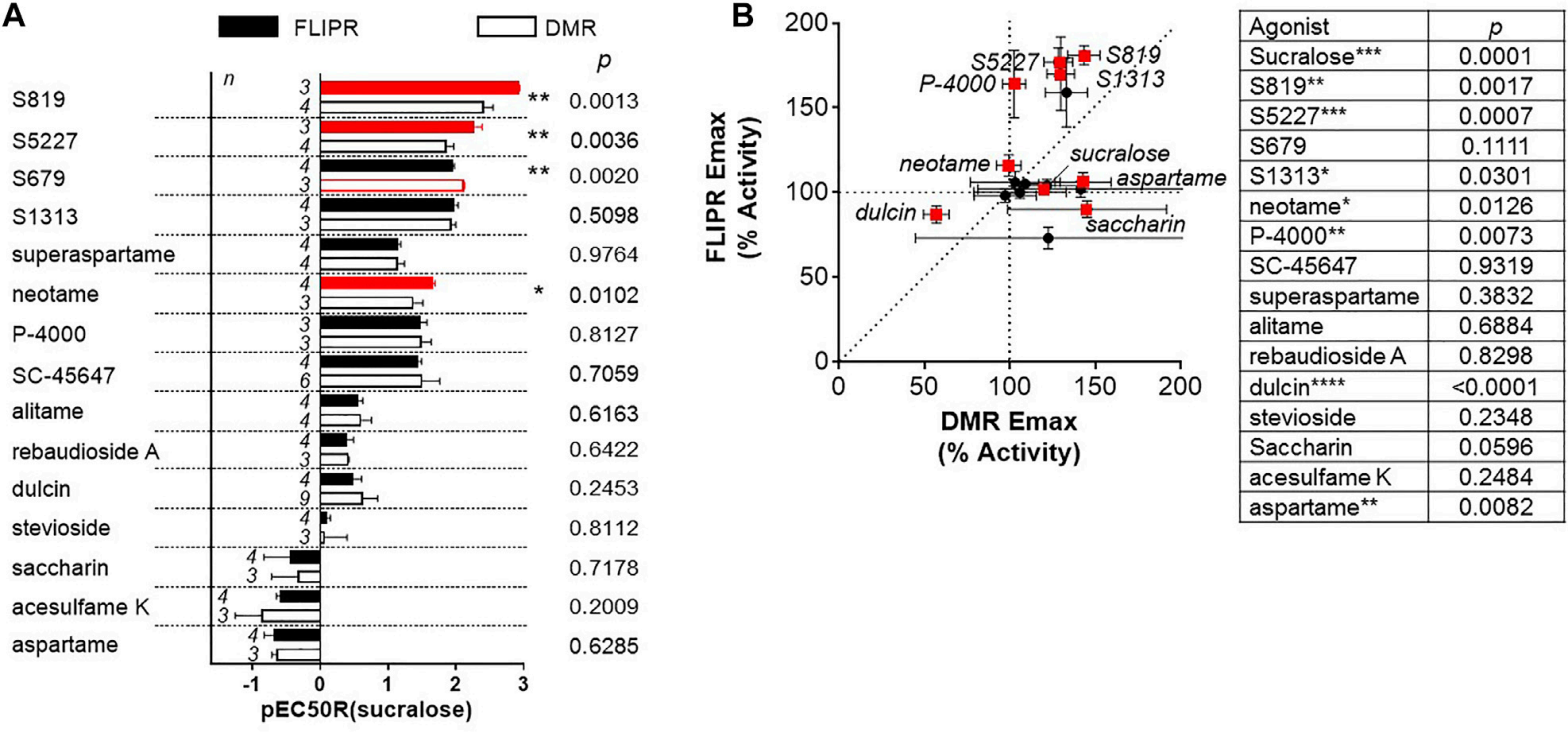
Potency and efficacy bias analysis. **(A)** Agonist potency values (pEC50s) obtained in each assay were normalized to that of sucralose as described in *Materials and Methods*. Each pair of the resulting values (pEC50R(sucralose)) were then analyzed by an unpaired, *t*-test (α = .05). **(B)** Agonist efficacy (Emax) values obtained in each assay were and summarized in Table 1 were plotted against each other. Each pair of Emax values were analyzed by an unpaired, *t*-test (α = .05) (***p < 0.001, **p < .01, *p < .05).

In the following analysis we directly compared the agonist activities measured in both assays using bias plotting. This approach allows the visualization of bias for an agonist in one assay versus another and has become an increasingly popular tool to identify and characterize biased agonists for GPCRs (Drake et al., 2008; Peters and Scott, 2009; Gregory et al., 2010; Thomsen et al., 2012; Kenakin, 2015; Montero-Melendez et al., 2015). Each agonist is tested at equimolar concentrations in both assays, the responses are normalized against a fixed concentration of a control agonist, and the resulting activity values obtained in one assay is plotted against the activity obtained in the other assay on the same graph. As shown in Figure 5, sucralose, saccharin. acesulfame K, SC-45647, rebaudioside A, stevioside, aspartame, alitame and superaspartame exhibited a similar slight bias towards the FLIPR assay as curves were of a comparable fit and almost superimposable (Figures 5H–O). Relative to sucralose and the other agonists, the bias curves for S679 and dulcin displayed a linear relationship that were also slightly biased towards the FLIPR assay (Figures 5F,G). S819, S5227, S1313, P-4000 and neotame were by far the most biased in this type of analysis (Figures 5A–E) in some instances reaching superagonist level of activity in the FLIPR assay at concentrations producing about 50% activity in the DMR assay (Figures 5A,B). To statistically quantify bias while accounting for changes in cell line sensitivity over time (since these experiments were run independently) we normalized each agonist pEC_50_ value to that of sucralose (ran on the same day) in both assays and derived a pEC_50_R (sucralose) value (see *Materials and Methods*), thereby minimizing the potential effect of daily assay sensitivity fluctuations. While most agonists displayed similar pEC_50_R (sucralose) values between assays, S819, S5227 and neotame had significantly higher pEC_50_R (sucralose) values in the FLIPR assay (Figure 6A). Statistical analysis of EC_50_ ratios also pointed to S679 as being slightly but significantly more potent in the DMR assay vs the FLIPR assay (Figure 6A) a conclusion that could not be supported by the bias plotting analysis performed in Figure 5. In addition, S819, S5227, P-4000, S1313, neotame and dulcin exhibited a statistically significant efficacy bias (see *Materials and Methods*) towards the FLIPR assay (Figure 6B) suggesting that part of these agonist bias observed in Figure 5 was also due to an increase in efficacy in the FLIPR assay vs the DMR assay. Conversely, sucralose, saccharin and aspartame apparently displayed a greater efficacy bias towards the DMR assay (Figure 6B). However, the higher Emax values for these lower potency agonists (such as saccharin and aspartame), is partly due to the poorly defined top asymptote of the dose-response DMR curves (Supplementary Figure S2). Ligand bias can also be estimated with the use of an operational model as described (Kenakin et al., 2012; Nagi and Pineyro, 2016). This approach takes into account the influence of both ligand affinity for the receptor, which is a potency-related parameter termed K_A_, and signaling efficacy, termed τ, and produces a transduction coefficient, termed log (τ/KA; also termed logR), consolidating functional affinity and efficacy information to estimate bias. Practically, fitting the dose-response curves to the model generates ligand and assay specific transduction coefficients. By subtracting each ligand specific logR values with that of a standard ligand (in our case sucralose) ran in the same assay (to take into account variations brought upon by the assays themselves), one obtains ΔlogR values for each ligand and assays and these can be used to estimate and statistically confirm bias. Subtracting the ligand specific ΔlogR values between assays provides ΔΔlogR values and the inverse log of these values in turn provides the magnitude of the bias or a bias factor. As shown in Table 2, fitting of the data to the operational model confirmed that S819, S5227 and neotame are indeed biased for the FLIPR assay and calculating the inverse log of the ΔΔlogR values produced bias factors varying between 3 and ∼5. The absolute ΔΔlogR values for P-4000 and S1313, 0.36 and 0.22, respectively, were not statistically different than 0 with the available data and standard errors. Operational bias analysis also indicated a potential small bias of alitame and acesulfame K for the FLIPR and DMR assays, respectively (Table 2).

**TABLE 2:** Transduction coefficients, ΔlogR and ΔΔlogR values obtained from dose-response FLIPR-Gα_15_ and DMR data fitting using the operational model. Ligand bias was estimated as described in *Materials and Methods*.

Agonist	ΔlogR FLIPR			ΔlogR DMR			ΔΔlogR				Bias factor
	Mean	Sem	*N*	Mean	Sem	*N*	Mean	Sem	Df	95% confidence interval	
S819	3.11	0.03	3	2.52	0.04	4	0.59	0.05	5	0.45 to 0.73	3.9
S5227	2.52	0.08	3	1.84	0.08	4	0.68	0.12	5	0.38 to 0.97	4.8
Neotame	1.65	0.03	4	1.15	0.04	3	0.50	0.05	5	0.37 to 0.67	3.2
P-4000	1.67	0.02	3	1.31	0.14	3	0.36	0.14	4	−0.04 to 0.75	
S1313	2.14	0.04	4	1.91	0.20	3	0.22	0.20	5	−0.29 to 0.74	
S679	2.15	0.03	4	2.07	0.15	3	0.08	0.15	5	−0.31 to 0.47	
Superaspartame	1.17	0.03	4	1.04	0.05	4	0.13	0.06	6	−0.01 to 0.27	
Alitame	0.52	0.04	4	0.35	0.03	4	0.18	0.05	6	0.05 to 0.31	1.5
SC-45647	1.45	0.02	4	1.38	0.10	6	0.07	0.10	8	−0.17 to 0.31	
Dulcin	0.38	0.06	4	0.20	0.05	9	0.18	0.08	11	0.00 to 0.36	
Rebaudioside A	0.38	0.05	4	0.22	0.15	3	0.16	0.15	5	−0.24 to 0.56	
Stevioside	0.13	0.01	4	0.09	0.12	3	0.04	0.13	5	−0.29 to 0.36	
Saccharin	−0.47	0.21	4	−0.31	0.09	3	−0.16	0.23	5	−0.74 to 0.42	
Aspartame	−0.74	0.05	4	−0.72	0.08	3	−0.03	0.10	5	−0.27 to 0.22	
Acesulfame K	−0.74	0.02	4	−0.99	0.09	3	0.26	0.09	5	0.01 to 0.50	1.8

**FIGURE 7:**
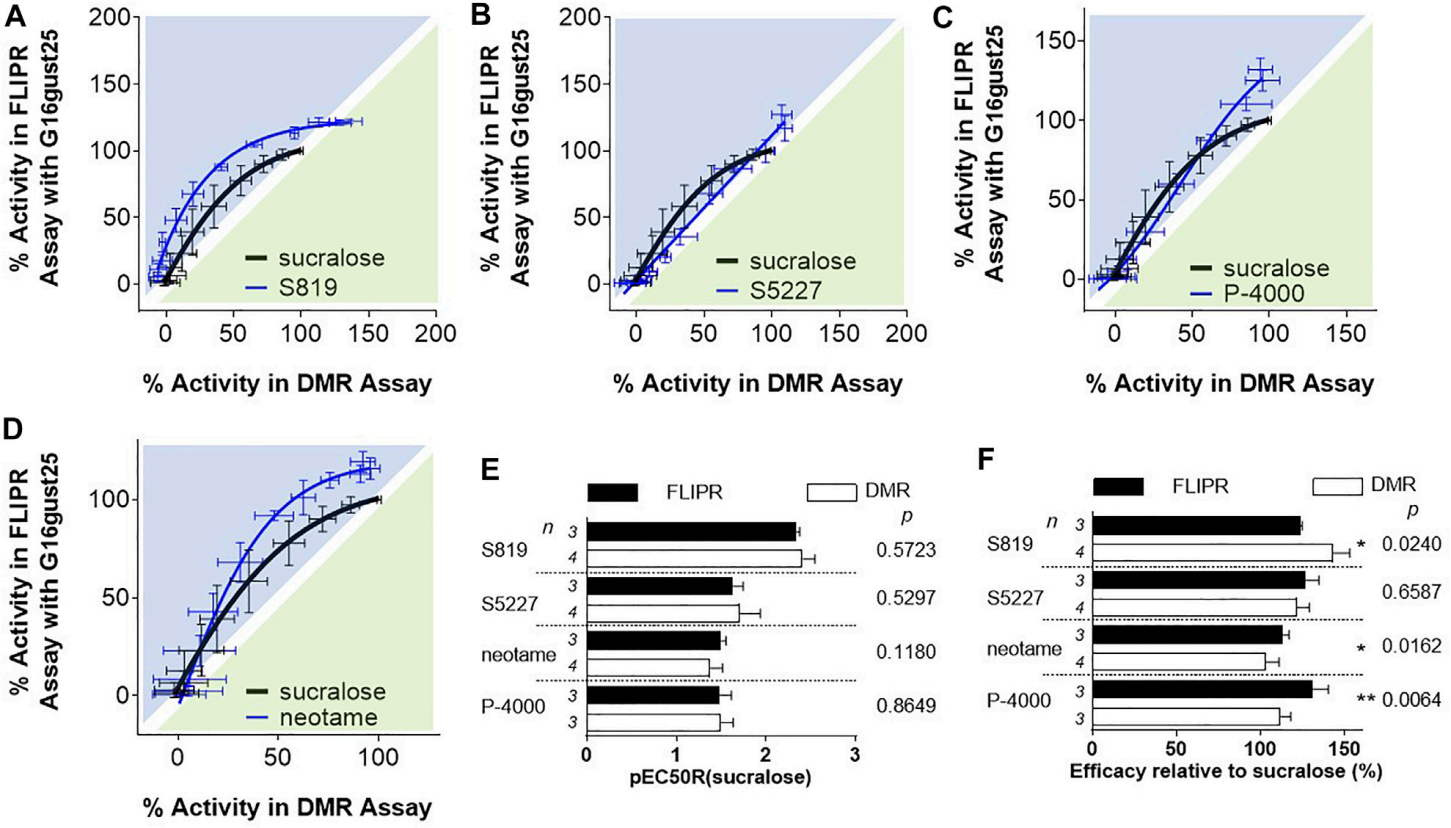
Evaluation of agonists in the FLIPR assay with R2/R3 U2OS cells expressing Gα_16gust25_. For Panels **(A**–**D)**, bias plotting analysis as described in Figure 5. Traces are representative of three independent experiments and data points correspond to an average and standard deviation of triplicate determinations. **(E)** Potency bias analysis was performed as described in Figure 6. **(F)** Efficacy bias analysis was performed as described in Figure 6.

Since the DMR and FLIPR assays use different G proteins to transmit intracellular signals, we next sought to determine if the apparent potency and/or efficacy bias for the FLIPR assay were a direct consequence of the coupling of the sweet taste receptor to the promiscuous G protein Gα_15_. The C-terminal residues of Gα proteins are in direct contact with GPCRs and they dictate coupling specificity (Conklin et al., 1993; Mody et al., 2000; Rasmussen et al., 2011). To assess the influence of the carboxyl terminal region of Gα_15_ on potency and to render Gα_15_ more Gα_i/o_-*like* we made a chimeric G protein where the last 25 C-terminal amino acids of Gα_16_ (the human orthologue of Gα_15_) were replaced with the last C-terminal 25 amino acids of Gα_gustducin_, a G protein belonging to the Gα_i/o_ family (McLaughlin et al., 1992), and that is absolutely required for sweet and bitter taste (Wong et al., 1996), and therefore thought to represent the physiologically relevant G protein for taste receptors. Remarkably, when the FLIPR assay was run in the context of Gα_16gust25_, bias plotting analysis uncovered that S819, S5227, P-4000 and neotame exhibited a noticeable decrease in bias for the FLIPR assay (Figures 7A–D and compare with Figure 5). Accordingly, these ligands did not show statistical differences in their pEC_50_R (sucralose) values (Figure 7E). In the context of Gα_16gust25_ S819, S5227, and P-4000 FLIPR efficacy values were decreased relative to the values obtained in the Gα_15_-based FLIPR assay (compare values in Figures 6B, 7F). However, P-4000 and neotame’s efficacy values were still significantly greater in the FLIPR assay while the S819 DMR efficacy value was greater in the DMR assay (Figure 7F). Noticeably, while the assay bias described in Figure 5 is clearly not strictly dependent on the type of assay per se (since the nature of the G protein clearly plays a significant role, as shown in Figure 7), it influences bias to a degree. As shown in Supplementary Figure S6, analysis of the FLIPR assay data run either with Gα_15_ or Gα_16gust25_ still shows S819, S5227, P-4000 and neotame bias (to a lower degree) towards the assay run with Gα_15_ as the promiscuous G protein. Of note, however, is the observation that the slight FLIPR assay bias detected for sucralose when comparing DMR and FLIPR assay data (Figures 5, 7) is abolished when comparing FLIPR assay ran with different G proteins (Supplementary Figure S6). Also, while the use of Gα_16gust25_ abolished S819, S5227 bias when using the operational model to estimate bias, noetame’s bias was only decreased with this G protein (Table 3). Collectively, these data demonstrate that the nature of the G protein and, to a point, the types of assays can affect the relative bias of sweet taste receptor agonists.

**TABLE 3:** Transduction coefficients, ΔlogR and ΔΔlogR values obtained from dose-response FLIPR-Gα_16gust25_ and DMR data fitting using the operational model. Ligand bias was estimated as described in *Materials and Methods*.

Sweetener	ΔlogR FLIPR			ΔlogR DMR			ΔΔlogR				Bias factor
	Mean	Sem	*N*	Mean	Sem	*N*	Mean	Sem	Df	95% confidence interval	
S819	2.40	0.09	3	2.52	0.04	4	−0.13	0.10	5	−0.37 to 0.12	
S5227	1.73	0.06	3	1.84	0.08	4	−0.12	0.10	5	−0.38 to 0.15	
Neotame	1.52	0.06	3	1.15	0.04	3	0.36	0.07	4	0.17 to 0.56	1.5

**FIGURE 8:**
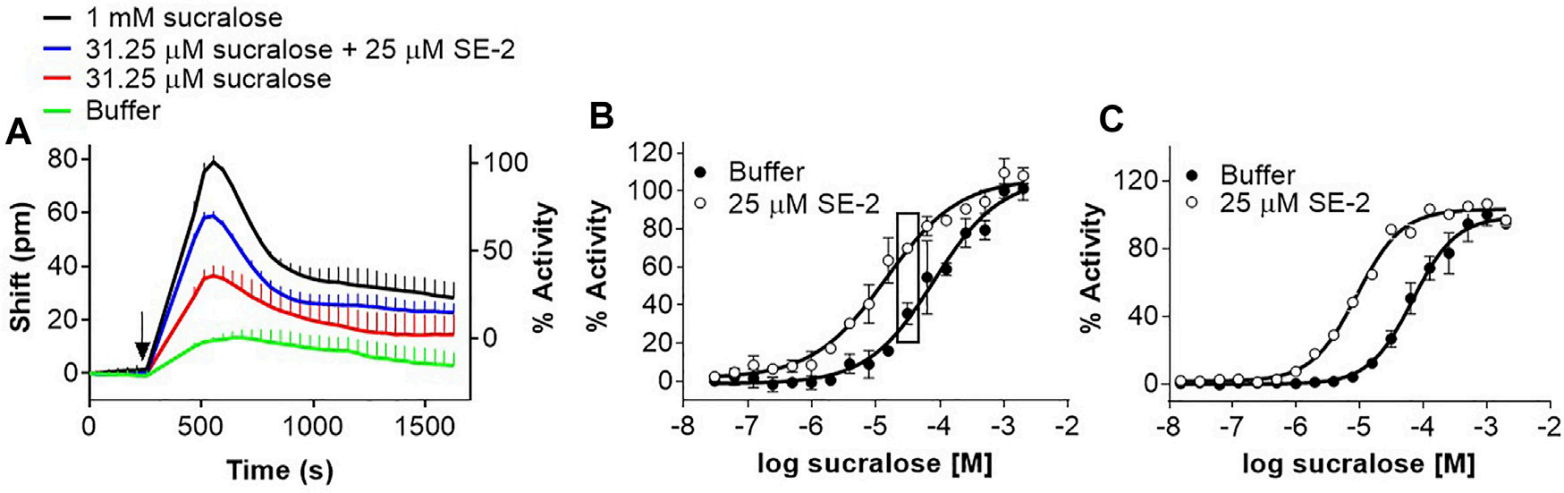
Evaluation of a PAM for the human sweet taste receptor in the DMR and FLIPR assays. **(A)** R2/R3 U2OS cells were stimulated with increasing concentrations of sucralose in the presence and absence of 25 μM SE-2 and DMR responses were monitored on the Epic^®^ reader. Depicted are the kinetics corresponding to the effect of SE-2 on 31.25 μM sucralose. **(B)** Depiction of the full sucralose dose-response analysis of the experiment described in **(A)**. The rectangles include data points from which the kinetics in Panel A were taken. **(C)** R2/R3 U2OS cells were transduced with Gα_15_ baculovirus. After 48 hours, cells were loaded with Fluo4 and stimulated with increasing concentrations of sucralose in the presence and absence of 25 μM SE-2 and responses were monitored on the FLIPR. Curves are representative of three independent experiments and data points correspond to an average and standard deviation of a triplicate determination.

We extended our investigation to other types of modulators. Positive allosteric modulators (PAMs) and negative allosteric modulators (NAMs) for the human sweet taste receptor have been identified or characterized with calcium mobilization cell-based assays using promiscuous G proteins. SE-2, a sweet taste receptor PAM (see structure in Figure 1), was identified using a Gα_15_-PLC cell-based assay (Servant et al., 2010; Servant et al., 2011). This PAM also enhanced the sucralose response in the DMR assay in U2OS cells (Figure 8A), producing a leftward shift in the dose-response of 5.1 ± 1.5-fold (mean ± sd, *n* = 3) (Figure 8B). As described previously (Servant et al., 2010; Servant et al., 2011), SE-2 did not produce any agonist activity on its own, as shown by the lack of activity at lower sucralose concentrations (Figure 8B). A slightly higher enhancement factor (7.3 ± 0.9-fold; mean ± sd, *n* = 4) could be calculated in the FLIPR assay on R2/R3 U2OS cells overexpressing Gα_15_ (Figure 8C).

**FIGURE 9:**
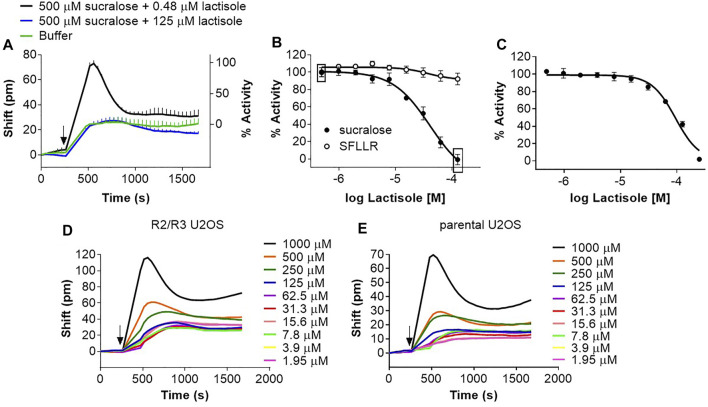
Evaluation of a NAM for the human sweet taste receptor in the DMR and FLIPR assays. **(A)** R2/R3 U2OS cells were stimulated with a fixed concentration of sucralose (500 μM) and with increasing concentrations of lactisole and DMR responses were monitored on the Epic^®^ reader. Depicted are the kinetics corresponding to the effect of 0.48 μM and 125 μM lactisole on the sucralose effect. **(B)** Depiction of the full lactisole dose-responses analysis of the experiment described in **(A).** The rectangle includes data points from which the kinetics in Panel A were taken. **(C)** R2/R3 U2OS cells were transduced with Gα_15_ baculovirus. After 48 hours, cells were loaded with Fluo4 and stimulated with a fixed concentration of sucralose (500 μM) with increasing concentrations of lactisole and responses were monitored on the FLIPR. Curves in **(B**,**C)** are representative of three independent experiments and data points correspond to an average and standard deviation of a triplicate determination. **(D)** Effect of increasing concentrations of lactisole on R2/R3 U2OS cells’ basal DMR response. Kinetics are representative of three independent experiments. **(E)** Effect of increasing concentrations of lactisole on parental U2OS cells’ basal DMR response. Kinetics are representative of three independent experiments.

Lactisole [the sodium salt of 2-(4-methoxyphenoxy)-propionic acid; Figure 1], an antagonist of the human sweet taste receptor (Xu et al., 2004), inhibits sweet receptor function in a manner that is either non-competitive or competitive depending on the agonist (Winnig et al., 2007; Servant et al., 2020). Moreover, lactisole also works as an inverse agonist by inhibiting the apparent constitutive activity of the sweet receptor (Galindo-Cuspinera et al., 2006). Lactisole inhibited the sucralose response in the DMR assay (Figure 9A). The effect of lactisole was dose-dependent with an IC_50_ of 38 μM (pIC50: 4.453 ± 0.206; mean ± sd, *n* = 4) (Figure 9B), and specific since other GPCR responses were not affected by lactisole (Figure 9B and results not shown). Similarly, lactisole inhibited the sucralose response in the FLIPR assay with an IC_50_ of 102 μM (pIC_50_: 3.994 ± 0.06; mean ± sd, *n* = 3) (Figure 9C). Characterization of inverse agonists has been performed using DMR. In one example, when added directly as stimuli on the cells, the dopamine receptor antagonists haloperidol and amisulpride *reversed* the kinetics from a classical positive DMR effect detected with an agonist to a negative DMR effect (Lee, 2009). We therefore reasoned that, on its own, lactisole could produce a negative DMR response in R2/R3 U2OS cells and that this effect should not be picked up with the parental cell line. However, we could not detect any evidence of constitutive activity for the human sweet receptor in the DMR assay. Increasing concentrations of lactisole (in the absence of agonist) did not change the basal DMR signal at up to 125 μM (Figure 9D) in R2/R3 U2OS cells, a concentration almost fully inhibiting an agonist effect (Figure 9B). At higher concentrations, lactisole produced a positive DMR response that could also be detected in the parental U2OS cell lines (Figures 9D,E) suggesting that this effect was non-specific.

## Discussion

### A New DMR Assay for the Human Sweet Taste Receptor

Over the last few years there has been an increased level of scrutiny regarding properties of GPCR modulators characterized or identified with cell-based assays. Terms such as *biased agonism* or *functional selectivity* have emerged to describe cases where ligands exhibit different activity profiles, affinity or efficacy, depending on the signaling pathway or the second messenger being measured, the cellular background, or the probe (probe dependence) (Galandrin et al., 2007; Kenakin, 2008; Kenakin, 2010; Kenakin, 2015; Montero-Melendez et al., 2015; Kenakin, 2019; Wingler and Lefkowitz, 2020). To evaluate functional selectivity in taste receptors we undertook the most comprehensive pharmacological characterization of human sweet taste receptor reported to date. We evaluated 19 agonists and other modulators using two different cell-based assays that were developed in the same cellular background. Instead of relying exclusively on coupling to promiscuous G proteins such as Gα_15_ (Nelson et al., 2001; Li et al., 2002; Bassoli et al., 2008; Li and Servant, 2008; Servant et al., 2010; Zhang et al., 2010; Bassoli et al., 2014), we developed an alternative assay using DMR which measures coupling to endogenous G protein and signaling pathways. This holistic approach allowed the detection of high potency agonists and allosteric modulators’ effects on the sweet receptor. The use of the DMR also allowed the identification of agonists with a potency and/or efficacy bias for the classical and more popular calcium mobilization FLIPR assay ran with Gα_15_.

A few technologies have been developed over the past several years that allow detection of the integrated phenotypic cellular response to a ligand without having to monitor the activation state of one particular signaling pathway. One of these technologies developed by Corning is an optical biosensor, called Epic^®^, which uses a resonant-waveguide grating for monitoring biomolecular interactions (Fang et al., 2006). Cells expressing receptors of interest are seeded onto the biosensor (the waveguide grated surface) and are illuminated with a polarized broadband light source. Modulators are added onto the cells and a shift in the wavelength of the reflected light is recorded. Typically, receptor modulators will produce either a positive DMR response, where movement of cellular components into a focal plane within ∼150 nm of the substrate increases the wavelength of the reflected light or, conversely, a negative DMR response where movement of cellular component outside the focal plane decreases the wavelength of the reflected light. Numerous GPCRs have been evaluated in label free platforms such as the Epic^®^ including the metabotropic acetylcholine receptors (Dodgson et al., 2009; Kebig et al., 2009; Lee, 2009; Schroder et al., 2010; Schroder et al., 2011; Schrage et al., 2013), dopamine receptors (Lee, 2009), cannabinoid receptors (Schroder et al., 2010; Codd et al., 2011), prostaglandin receptors (Schroder et al., 2010), free fatty acid receptor (Schroder et al., 2010; Schmidt et al., 2011), adrenergic receptors (Schroder et al., 2010; Ferrie et al., 2014; Grundmann et al., 2018), γ-aminobutyric acid receptors (Klein et al., 2016), the nociceptin/orphanin FQ peptide receptor (Malfacini et al., 2018), the neuropeptide S receptor (Ruzza et al., 2018), the uracil nucleotide/cysteinyl leukotriene receptor (Grundmann et al., 2018), histamine receptors (Seibel-Ehlert et al., 2021), the urotensin receptor (Lee et al., 2014) and opioid receptors (Codd et al., 2011; Morse et al., 2011). It has now become clear that both the cellular context and the type of coupling can directly influence the kinetics (positive or negative DMR) of a modulator in such a cell-based assay (Schroder et al., 2010; Schroder et al., 2011). However, up to now, none of the taste receptors had been evaluated in this platform.

In the DMR assay data presented here, activation of the sweet taste receptor expressed in U2OS cells led to a positive and bi-phasic DMR signal with 16 of the agonists we evaluated. Effects were dose-dependent and could not be detected on the parental cell lines at concentrations up to 1–2 mM. EC_50_ values confirmed that, overall, agonists activated the sweet taste receptor with the expected rank order of potency based on human taste data (Schiffman and Gatlin, 1993; Li and Servant, 2008; Palmer et al., 2021). At concentrations ≥3 mM, we could detect receptor independent positive DMR responses. Noticeably, low potency carbohydrate sweeteners such as sucrose and fructose exhibited a high degree of interference with the sensor precluding their characterization in the DMR assay. A NAM and a PAM for the human sweet taste receptor were evaluated in the DMR assay and their potency (IC_50_ for lactisole) or efficacy (magnitude of the dose-response shift produced by SE-2) were similar to the values obtained on the FLIPR assay. It has been previously reported that the DMR assay can detect the activity of GPCR inverse agonists (Lee, 2009). However, we could not detect the inverse agonist effect of lactisole, first reported in a calcium mobilization assay, (Galindo-Cuspinera et al., 2006), in our new DMR assay for the human sweet taste receptor. It is possible that U2OS cells adapt to the increased level of sweet taste receptor activity and that, therefore, no effect on DMR can be detected. Alternatively, a lower receptor density in our R2/R3 U2OS stable cell line could prevent detection of the constitutive activity of the human sweet taste receptor.

Using specific pathway blockers and toxins we discovered that the sweet taste receptor-mediated DMR responses observed in U2OS cells were mediated by Gα_i/o_ proteins, as highlighted by the inhibitory effect of PTx treatment. This observation agrees with published sweet taste receptor G protein coupling selectivity data. Notably, more than 25 years ago, studies in knock-out mice showed that Gα_gustducin_, a member of the G_i/o_ family of G proteins (McLaughlin et al., 1992), is absolutely required for behavioral and taste nerve responses to sweeteners (Wong et al., 1996). Furthermore, studies performed with sweet taste receptor subunits expressed in heterologous cells showed PTx-sensitive coupling to endogenous G_i/o_ proteins, leading to inhibition of cAMP accumulation and activation of MAPK (Ozeck et al., 2004). Sweet taste receptor subunits expressed in insect cell membranes can activate Gα_transducin_, Gα_i1_, Gα_o_ but not Gα_q_ and Gα_s_ (Sainz et al., 2007). Collectively, these results confirm that the sweet taste receptor naturally and preferentially couples to members of the G_i/o_ proteins *in vivo* and *in vitro*. Among the Gα_i/o_ members, Gα_i2_ is found at the highest level in U2OS cells, followed by Gα_i3_ and Gα_i1_, that are expressed at 5- to 10-times lower levels, while Gα_transducin_ and Gα_gustducin_ are either barely detectable or absent at the mRNA level (Uhlen et al., 2015; Karlsson et al., 2021). It is therefore likely that the human sweet taste receptor DMR responses are mediated in large part *via* activation of Gα_i2_ in U2OS cells. An intact actin cytoskeleton was also required for optimal DMR responses of every agonist studied, including those for endogenously expressed receptors in U2OS cells. Similarly, β2-adrenergic (Gα_s_-mediated) (Ferrie et al., 2014) and cannabinoid (Gα_i/o_-mediated) (Codd et al., 2011) DMR responses were abolished by latrunculin treatment.

To our knowledge, the signaling pathways downstream of Gαi/o proteins and required for DMR responses have not been identified, as most of the studies have limited their investigation to the use of PTx and no other pathway blockers. We therefore attempted to identify the signaling pathways contributing to the DMR response that lie downstream of Gα_i/o_ proteins and that are linked to actin cytoskeleton remodeling following sweet taste receptor activation in U2OS cells. Inhibitors of PI3K and MAPK, two major signaling components of the actin remodeling signaling machinery that are activated by Gα_i/o_ coupled GPCRs (Rickert et al., 2000), failed to inhibit the sucralose-mediated DMR responses while they significantly inhibited the EGF-mediated responses. In agreement, U0126 failed to inhibit the μ opioid receptor PTx-dependent DMR response in CHO cells (Codd et al., 2011) and wortmannin abolished the EGF-mediated DMR responses in UPCI-37B SCCHN cells (Du et al., 2009). Similarly, inhibitors of PKC and ROCK, other signaling molecules promoting receptor-mediated actin polymerization and stress fiber formation (Mackay and Hall, 1998; Rickert et al., 2000), could not inhibit the sucralose-mediated DMR responses but they respectively inhibited the carbachol and S1P-mediated DMR responses. Carbachol exerts its effects through either Gα_q_-coupled receptors (M1, M3, and M5) or Gα_i_-coupled receptors (M2 and M4) (Caulfield and Birdsall, 1998). That PTx failed to inhibit carbachol responses (Figure 3) suggests this agonist activates Gα_q_-coupled receptors in U2OS cells, ultimately resulting in PKC activation. S1P also activates five different receptors that couple to either Gα_i/o,_ Gα_q_, Gα_12/13_, and Gα_s_ proteins (Chun et al., 2010). The absence of a significant effect of PTx and our results with the ROCK inhibitor suggest that at least one of the Gα_12/13_-coupled receptors (S1P_2_, S1P_4_ or S1P_5_) is responsible for the DMR responses observed in U2OS cells. Finally, preventing a potential EGFR transactivation with specific antagonists did not preclude the sucralose-mediated DMR responses but fully inhibited the EGF-mediated response. Thus, additional work would be necessary to identify the effectors downstream of the human sweet receptor and Gα_i/o_ proteins that are responsible for the positive DMR responses in U2OS cells. The Gβγ subunit of activated Gα_i_ proteins is a potential candidate since it has been shown to directly trigger specific exchange factors which in turn activate the Rho GTPase Cdc42 leading to actin remodeling (Meili and Firtel, 2003; Ueda et al., 2008; Yan et al., 2012). In support of this hypothesis, a recent study shows that inhibition of the βγ-arm of the histamine h4R signaling pathway decreases DMR responses (Seibel-Ehlert et al., 2021).

Together, these results show that the new DMR assay for the human sweet taste receptor can measure effects of high potency agonists, PAMs and NAMs through recruitment of endogenously expressed Gα_i/o_ proteins and unidentified downstream effectors. This is in contrast to the classical use of overexpressed promiscuous G proteins to measure taste receptor activation in cell-based assays *via* a targeted PLC activation (Chandrashekar et al., 2000; Nelson et al., 2001; Bufe et al., 2002; Li et al., 2002; Nelson et al., 2002; Xu et al., 2004). On the other hand, the incompatibility of carbohydrate agonists with the DMR assay may limit its application to higher potency agonists or modulators. Moreover, the lower throughput and increased variability (comparing standard deviation on activity and pEC_50_ values in Table 1; Figure 4) make the DMR assay more suited as a secondary screening platform to further profile new leads.

### Sweet Receptor Agonists Exhibit Functional Bias in Cell-Based Assays

Functional bias is usually determined upon the comparison of the behavior of a receptor’s natural ligand versus the behavior of its synthetic ligands in two or more different assays (Stallaert et al., 2011). In our investigation, obvious natural ligands for the sweet taste receptor such as the carbohydrate sweeteners sucrose and fructose were incompatible with the DMR assay (Supplementary Figure S4 and results not shown). We therefore performed our bias plot analysis using sucralose, a structurally close analogue of sucrose (Figure 1). The bias plots indicated that all agonists had at least a slight preference towards calcium mobilization relative to the DMR response. However, not all agonists exhibited the same level of bias. Out of the sixteen agonists evaluated, eight agonists displayed superimposable curves with that of sucralose. These agonists included, saccharin, aspartame, superaspartame, alitame, SC-45647, the natural steviol glycosides rebaudioside A and stevioside and finally acesulfame K. Accordingly, these agonists did not exhibit significant changes in their relative potency to that of sucralose [pEC_50_R(sucralose) values] between the two assays and only two of these agonists, alitame and acesulfame K, exhibited small and barely significant bias following analysis with the operational model. Bias for the FLIPR assay could be explained with agonists exhibiting both a potency and efficacy bias, such as the thiourea S819, its analogue S5227, and the agonist neotame. Accordingly, these molecules exhibited the greatest level of bias following analysis with the operational model. On the other hand, the bias of agonists exhibiting exclusively an efficacy bias, such as S1313 and P-4000 could not be confirmed with the operational model. It is possible that the bias, clearly detected by bias plotting is either too low to be detected by fitting the dose-response data to an operational model and/or that the experimental error is too high to achieve significance. The agonists sucralose, saccharin and aspartame apparently exhibited a greater efficacy bias towards the DMR assay. However, this is likely due in part to the difficulty in obtaining defined dose-response top asymptotes for these sweeteners in the DMR assay. Potency (EC_50_) values of sweet taste receptor agonists are known to vary by 2- to 10-folds from laboratory to laboratory and with different experimental conditions (Palmer, 2019; Servant et al., 2020; Ahmad and Dalziel, 2020; Belloir et al., 2021). The potency values reported in this study fall within this range. However, to minimize the impact of this inherent fluctuation, we conducted our experiment in the same cellular background, used the same sweet taste receptor stable clone between assays and normalized cell responses to an internal control (sucralose) in both assays. Importantly, the observation that only selected sweet taste receptor agonists exhibited a markedly enhanced bias for the FLIPR assay, as opposed to all agonists studied, ruled out the possibility that the observed bias could be merely due to comparison of two assays not expressing the same level of G proteins (over-expressed Gα_15_ vs. endogenously expressed Gα_i/o_ proteins).

Our investigation also highlighted that the potency of sweet receptor agonists, such as S819, S5227, P-4000 and neotame, can be influenced by the nature of the G protein coupling. Experiments performed with the chimeric G protein Gα_16gust25_ suggest that the C-terminal coupling residues of the Gα subunit are responsible for the relatively higher level of bias of these agonists for the FLIPR assay. Indeed sucralose, S819, S5227, P-4000 and neotame exhibited nearly superimposable bias plots when the FLIPR assay was run with Gα_16gust25_. Our data therefore suggests that different binding domains of the sweet taste receptor seem to be influenced allosterically by the type of G protein. S819 binds to the transmembrane domain (TMD) of hT1R2 to elicit activation (Zhang et al., 2008) as does the G protein (Xu et al., 2004). Intuitively, it is plausible that G protein interaction at the cytosolic side of the hT1R2 TMD would influence a binding pocket located within its heptahelical domain. Neotame, however, binds to the Venus flytrap domain (VFD) of hT1R2 to elicit activation (Xu et al., 2004). Thus, G protein influence may be transmitted not only close to the G protein interacting site but also distally, to the outermost binding domain of the sweet taste receptor. Our study points to structure-dependent bias within classes of sweet taste receptor agonists. Neotame is a close analog of the sweet taste receptor agonists aspartame, alitame and superaspartame (see structures in Figure 1). Yet, neotame is the only agonist among this family to exhibit bias for the FLIPR assay. Other GPCR agonist responses are known to be directly and significantly influenced by G protein coupling and the rank order of agonist potency has been shown to change with different G proteins (Galandrin et al., 2007). Of particular relevance to our study, some olfactory receptor ligands exhibited good potency and efficacy in the context of Gα_15_ but were far less potent or totally inactive when evaluated in the context of Gα_olf_, the physiologically relevant G protein α-subunit for olfactory receptors (Shirokova et al., 2005). Furthermore, octanoic acid, a potent agonist of the olfactory receptor Ors6 in the context of Gα_15_ became an antagonist in the context of Gα_olf_ (Shirokova et al., 2005). In another study performed with a mutated M3 muscarinic receptor expressed in yeast, a modulator (brucine) was shown to behave either as a partial agonist with enhancement activity, a positive allosteric modulator without agonist activity or a neutral modulator (without any activity) depending on which G protein was expressed (Stewart et al., 2010). In a FLIPR assay, the rank order or potency between eel calcitonin and porcine calcitonin on the calcitonin receptor-2 was reversed when Gα_s_ was co-expressed (Watson et al., 2000). More recently, peptide analogues of oxytocin were shown to activate exclusively certain members of the Gα_i/o_ family members (Gα_i2_, Gα_i3_) while the natural ligand could activate all the family of Gα_i/o_ proteins evaluated (Gα_i1_, Gα_i2_, Gα_i3_, Gα_oA_, and Gα_oB_) *via* the oxytocin receptor (Busnelli et al., 2012).

Our results reveal that the DMR assay for the human sweet taste receptor can be an effective tool to characterize effects of potent agonists or other modulators through coupling to endogenous signaling pathways. Overexpression of promiscuous G proteins such a Gα_15_, may bias the effect of certain agonists in a FLIPR assay by overestimating their relative potencies and/or efficacies thereby potentially limiting the predictive nature of the assay.

## Data Availability

The original contributions presented in the study are included in the article/Supplementary Materials, further inquiries can be directed to the corresponding author.
