# Supplementary material for: A Dynamic Mass Redistribution Assay for the Human Sweet Taste Receptor Uncovers G-Protein Dependent Biased Ligands
Source: Front Pharmacol. 2022 Feb 17;13:832529. doi: 10.3389/fphar.2022.832529 (PMC8893300; doi:10.3389/fphar.2022.832529)
Supplement: Supplementary file 1 [file DataSheet1.docx]

Supplementary Material Servant et al.

**Supplementary Figure 1.** Representative effects of different pathway blockers and toxins on DMR kinetics of receptor agonists in R2/R3 U2OS cells.

**Supplementary Figure 2**. Representative non-normalized dose-response curves of the depicted agonists obtained in the DMR assay. Each point corresponds to an average and standard deviation of a triplicate determination.

**Supplementary Figure 3**. Representative non-normalized dose-response curves of the depicted agonists obtained in the FLIPR assay. Each point corresponds to an average and standard deviation of a triplicate determination.

**Supplementary Figure 4**. Effect of sucrose in the DMR assay. **(A)** Representative kinetics of 100 mM sucrose. **(B)** corresponds to a magnification of Panel **(A)**. **(C)** Representative kinetics of 30 mM sucrose. **(D)** corresponds to a magnification of Panel **(C)**. **(E)** Representative kinetics of 10 mM sucrose. **(F)** Representative kinetics of 3 mM sucrose. Kinetics correspond to an average and standard deviation of 24 wells treated as described on the graphs. **(G)** Bar graph summary of experiments depicted in **(A)** through **(F)**. Data are representative of 3 independent experiments. **(H)** R2/R3 USOS cells data depicted in Panel **(G)** was divided by the DMR responses observed on the parental U2OS cells and the quotient was multiplied by 100. **(I)** R2/R3 USOS cells data depicted in Panel **(G)** were subtracted by the DMR responses observed on the parental U2OS cells. *NS*, non-significantly different than 0, one sample t-test, *p* > 0.05.

**Supplementary Figure 5**. Evaluation of sucralose, fructose and sucrose in the FLIPR assay.

**Supplementary Figure 6**. Bias plotting of agonist dose-response curves obtained the FLIPR assay ran with cells expressing the sweet taste receptor and with Gα_15_ or with Gα_16gust25_.
